# Synergistic Effects of Exercise Training and Vitamin D Supplementation on Mitochondrial Function of Cardiac Tissue, Antioxidant Capacity, and Tumor Growth in Breast Cancer in Bearing-4T1 Mice

**DOI:** 10.3389/fphys.2021.640237

**Published:** 2021-04-13

**Authors:** Ali Jafari, Dariush Sheikholeslami-Vatani, Farnoosh Khosrobakhsh, Neda Khaledi

**Affiliations:** ^1^Department of Physical Education and Sport Sciences, University of Kurdistan, Sanandaj, Iran; ^2^Department of Cellular and Molecular Biology, Faculty of Science, University of Kurdistan, Sanandaj, Iran; ^3^Physical Education and Sports Sciences College, Kharazmi University, Karaj, Iran

**Keywords:** breast cancer, cardiovascular disease, exercise, vitamin D, synergistic effect, mitochondrial function

## Abstract

Both regular exercise training and vitamin D consumption are beneficial for patients with cancer. The study investigated the effects of interval exercise training (IET) or/and vitamin D supplementation on the gene expression involved in mitochondrial function of heart tissue, tumor size, and total antioxidant capacity (TAC) in breast cancer (BC) model mice. We assigned random 40 female NMRI mice to five equal groups (*n* = 8); the healthy control group (H.C), cancer control group (Ca.C), cancer with the vitamin D group (Ca.VD), cancer exercise group (Ca.Ex), and cancer exercise along with the vitamin D group (Ca.Ex.VD). Forty-eight hours after treatment, we anesthetized the animals and performed the isolation of heart tissue and blood serum for further studies. The results showed that the lowest mean body weight at the end of the treatments was related to Ca.C (*p* = 0.001). Vitamin D treatment alone has increased tumor volume growth by approximately 23%; in contrast, co-treatment with exercise and vitamin D inhibited tumor growth in mice (*P* = 0.001), compared with the cancer control (12%). TAC levels were higher in the group that received both vitamin D and exercise training (Ca.Ex.VD) than in the other treatment groups (Ca.VD and Ca.Ex) (*p* = 0.001). In cardiac tissue, vitamin D treatment induces an elevation significantly of the mRNA expression of *Pgc1−*α, *Mfn-1*, and *Drp-1* genes (*p* = 0.001). The study has shown the overexpression of vitamin D in female mice, and synergistic effects of IET with vitamin D on weight loss controlling, antitumorigenesis, improvement of antioxidant defense, and the modulation of gene expression. The synergistic responses were likely by increasing mitochondrial fusion and TAC to control oxidative stress. We recommended being conducted further studies on mitochondrial dynamics and biogenesis focusing on risk factors of cardiovascular disease (CVD) in patients with BC.

## Introduction

Female breast cancer (BC) has outstripped lung cancer as the most generally detected cancer, with an assessed 2.3 million new cases (11.7%), followed by lung (11.4%) and other cancers in 2020 (Sung et al., 2021). Because of progress in the prevention, untimely diagnosis, and treatment, early mortality of BC has diminished by almost 40% in the last four decades (Miller et al., 2018). Nevertheless, the risk of cardiovascular disease (CVD) mortality is remarkably increased following BC detection, and it is a main cause of death in this society (Kirkham et al., 2019). One of the other side effects of BC includes mitochondrial dysfunction, which is a hallmark of many diseases and plays a critical role in CVD pathogenesis (Maycotte et al., 2017; No et al., 2020), tumor cells (Gottlieb and Tomlinson, 2005; Kim et al., 2006), and cardiovascular disorders (Haas, 2019). Peroxisome proliferator-activated receptor γ coactivator-1 (PGC-1) is one of the genes involved in mitochondrial function which regulates numerous transcription factors responsible for energy metabolism and cardiac function, controlling mitochondrial biogenesis and dynamics, and modulating reactive oxygen species (ROS) homeostasis under physiological and pathological conditions. Moreover, cardiac PGC-1 coactivators appear to exert cardioprotective effects (Di et al., 2018; Wu et al., 2019). An animal model study reported that PGC-lα straightly controls Mfn1 gene transcription by coactivating the estrogen-related receptor α (ERRα) on a conserved DNA element. Surprisingly, PGC-1α deficit in the adult heart did not lead to evidence of uncommon mitochondrial dynamics (fusion and fission) or heart failure (Martin et al., 2014). The main role of Mitofusins for cardiac physiology was recently indicated, where genetic upset of Mfn1 leads to the storage of fragmented mitochondria and results in lethal heart failure in mice (Papanicolaou et al., 2012). A common feature of changed cells with dysfunctional mitochondria is elevated ROS generation (Ralph et al., 2010). ROS over-production causes a decrease of antioxidant enzymes and the failure of the cardiovascular system via disorder of downstream pathway the subcellular organelles. Abundant ROS may reduce nitric oxide (NO) bioavailability and NO/cGMP-dependent pathway which leads to pathological vasoconstriction and hypertension (Naseem, 2005).

Numerous studies have shown a positive effect of physical activity on BC, although no definitive conclusion has been reached (Westerlind et al., 2002; Malicka et al., 2015; Alvarado et al., 2016; Faustino-Rocha et al., 2016, 2017; Sturgeon et al., 2017). A recent study showed that a combination of treatments of high-intensity interval training (HIIT) and saffron supplementation affect weight loss and cachexia control in mice BC modeling (Ahmadabadi et al., 2020). As well, the next study reported that suppressed PGC-1a exhibits lower treadmill-running capacity and diminished cardiac function after exercise in mice (Lehman et al., 2000). Exercise also diminished the accumulation of MFN1 in the failing heart (Campos et al., 2017). Exercise increases the antioxidant defense mechanisms via redox-sensitive transcription factors, NF-κB and activator protein-1, and PGC-1α (Tsukiyama et al., 2017). Nevertheless, the capacity of exercise to impact PGC-1α in cardiomyocytes requires further investigation before this mechanism can be dismissed. By exercise training, enzymes generating ROS like p67phox protein diminish, and the enzymes participating in scrubbing oxygen-free radicals, such as superoxide dismutase (SOD) 1, SOD2, catalase, and glutathione peroxidase (GPX), are normalized and even improved (Oharomari et al., 2015). That means that all the proteins participating in oxidative stress are prone to reversal to normal levels after exercise conduct. The most extensively confirmed mechanism by which exercise training may arrest cardiotoxicity is through its antioxidant effects. The increase of antioxidative enzymes linked to exercise training may thus promote NO bioavailability by inhibiting ROS (Gielen et al., 2010). Other studies have shown that in animals that are physically active, the risk of acquiring diseases is decreased, possible tumor development is delayed, and smaller-sized tumors are observed (Malicka et al., 2015; Alvarado et al., 2016; Faustino-Rocha et al., 2016, 2017; Sturgeon et al., 2017).

Several of the investigations have reported which vitamin D intake and its higher circulating rates likely are protective against cancer and cardiovascular events (Atoum and Alzoughool, 2017; Jeon and Shin, 2018; Wimalawansa, 2019). In contrast, a study reported that intake of vitamin D did not lead to a less incidence of invasive cancer or CVD (Manson et al., 2018). As well, the studies in animals confirm mechanisms whereby vitamin D likely prevents carcinogenesis and decelerates tumor progression (Ross, 2011; Feldman et al., 2014). Mechanistic investigations have demonstrated that vitamin D impacts inflammatory proceeding involved in cancer development, including cytokines, prostaglandins, MAP kinase phosphatase 5 (MKP5), the nuclear factor kappa B (NF-κB) pathway, and immune cells (Raymond et al., 2014; Han et al., 2015). As well as another study confirmed that the vitamin D has the potential to prevent tumor progression by intermitting the inflammatory system (Liu et al., 2018).

Changes in cancers are dependent on a high-quality mitochondrial lake. Mitochondrial condition is regularly evaluated and properly better-quality by mitochondrial dynamics (cycles of mitochondrial fusion and division). New progress inconsiderate of mitochondrial dynamics in cancers has revealed its critical meaning in tumor growth and metastasis (Weiner-Gorzel and Murphy, 2021). Moreover, given with reports associated with the contradictory role(s) of levels of PGC1α expression linked to carcinogenesis in the early stage, and elevating tumor cell phenotype in the late stage (Mastropasqua et al., 2018), we derive that PGC1α is neither friend nor foe in cancers; due to importance of mitochondrial function for heart health and the controversial role(s) of PGC1α, antioxidant status, and cardioprotective effect of vitamin D, it is warranted to investigate PGC1α as considered main regulators of mitochondrial biogenesis and quality, and antioxidant potential effects in cardioprotection following interval exercise training (IET) along with vitamin D intake in BC. Here we characterize the impact of IET and vitamin D supplementation, a safe strategy against BC and CVD, on cardiac and its contribution to mitochondrial quality control, biogenesis, and total antioxidant capacity (TAC) in a post-tumorigenesis animal model.

## Materials and Methods

### Animal Handling and Research Process

**FIGURE 1:**
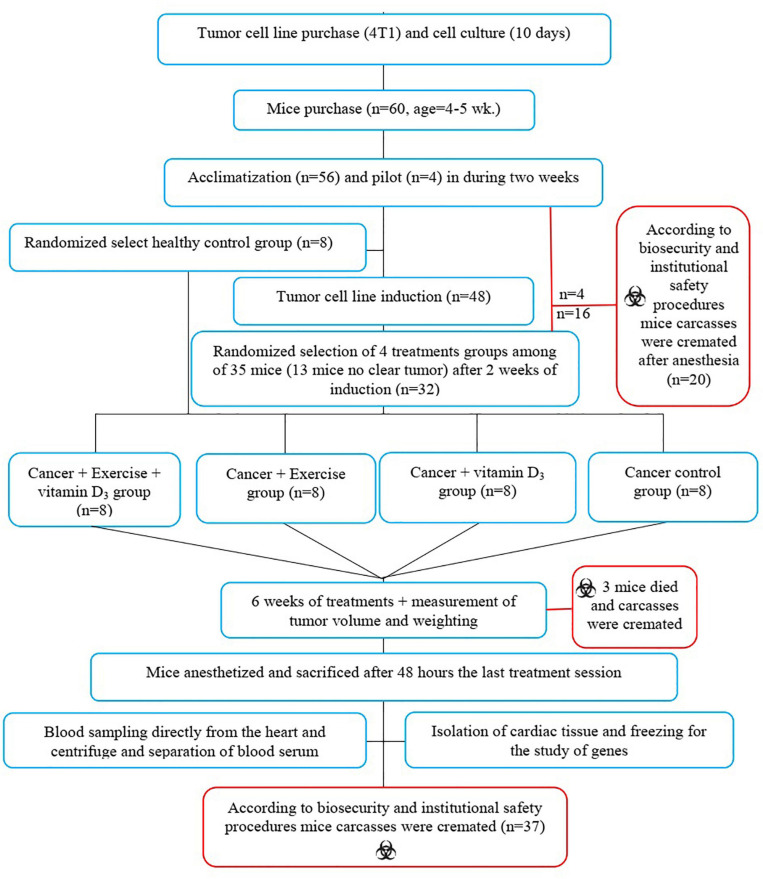
Study flow diagram and follow-up of the stages.

Sixty female NMRI mice weighing 24 ± 2 g and aged 4–5 weeks were purchased from Razi Vaccine and Serum Research Institute, Iran. Mice were housed at the Kharazmi University Animal Laboratory on Polycarbonate Cages under controlled environmental conditions (12/12 h light/dark cycle, TM; 23°C, HM; 42°) with free access to food and water. After 2 weeks of familiarization with the environment, eight mice were isolated as healthy control (H.C) and kept in the same conditions as the other animals until the end of the study period without any treatment. Four mice piloted to determine the best dose to induce BC among the remaining 52 mice. After determining the appropriate dosage, the remaining 48 mice were injected with a BC induction agent. Cancerous tumors were seen 2 weeks after injection. However, no clear BC tumors were observed in 12 animals and they were excluded from the study design. Then, 36 animals with BC (along with eight mice in the healthy control group) were randomly divided into the five groups: (I) healthy control (H.C), (II) cancer control (Ca.C), (III) cancer with vitamin D supplementation (Ca.VD), (IV) cancer with exercise training (Ca.Ex), and (V) cancer with exercise training and vitamin D supplementation (Ca.Ex.VD). It should be noted that two mice from the Ca.Ex.VD group (six mice remained) and one of the Ca.Ex group (seven mice remained) died during the treatment. Next, the animals in the treatment groups (U.Ca.VD, Ca.Ex, and Ca.Ex.VD) received the desired treatments (vitamin D supplementation, exercise training, or a combination of vitamin D with exercise training, respectively) for 6 weeks; 48 h after the last exercise training bout, the animals were sacrificed by intraperitoneal administration of 20–30 mg/kg ketamine 10% and 2–3 mg/kg xylazine 2%. Body weight was recorded weekly during the study (Figure 1). The present study was conducted according to the biosafety guidelines of the World Health Organization regarding laboratory animals and approved by the university’s biosafety committee (Approval ID: IR.UOK.REC.1397. 026).

### Cell Culture and Induction of Moue Model Breast Cancer

The mouse BC cell (4T1; NCBI No: C604) was obtained from the cell bank of Pasteur Institute of Iran. Cell lines were initially cultured with DMEM^1^, 10% fetal bovine serum (FBS), 1% penicillin-streptomycin, and non-essential amino acids at 37°C in a humidified atmosphere containing 5% CO_2_. Tumor cell suspension with > 90% viability was prepared from subconfluent cultures by the treatment of trypsin-EDTA solution (Invitrogen). Mice in cancer groups were subcutaneously injected with 1 × 10^5^ 4T1 cells into the right flanks, and sterilized distilled water with the same volume was injected subcutaneously into a healthy control group (Ya et al., 2015). To determine the optimal dose for tumor induction, cell suspensions in four different concentrations (1 × 1^4^, 1 × 1^5^, 5 × 1^5^, and 1 × 1^6^ cells per mouse) were injected subcutaneously to four healthy NMRI mice following the administration of local anesthetic in the right rear flank. The best-induced dose for BC was considered 1 × 1^5^ in the present study by observing neuromuscular symptoms, as well as the rate of weight loss in animals.

### Interval Exercise Training Protocol and Vitamin D Supplementation

Interval exercise training was performed for 6 weeks, three sessions per week, on a rodent treadmill (Danesh Salar of Iranian Co., IRI) in the evening (from 5:00 PM to 7:00 PM). Training intensity (running speed on the treadmill) and duration gradually increased every 2 weeks for the training groups. Due to the running speed and treadmill slope (zero degree), the intensity of training in the present protocol was equal to 50–75% of maximal oxygen consumption (VO_2_ max) in mice (Wolin et al., 2012). The rest period between exercise bouts was considered 1:4 (Table 1).

**TABLE 1:** Interval exercise training protocol.

**IET periodization**	Speed* (m/min)	**Duration (set × min)**	**Rest between workouts (min:s)**
Acclimatize IET 2 weeks	5–15	2 × 10	2:30
The first 2 weeks IET	20	2 × 12	3
The second 2 weeks IET	25	3 × 10	2:30
The third 2 weeks IET	20	4 × 12	3

*Grade (%) of treadmill was zero. IET, interval exercise training.

Previous studies have confirmed the anti-cancer effects of vitamin D (Wolin et al., 2012; Atoum and Alzoughool, 2017). In the current study, vitamin D (Sun Vit^®^, Iran Hormone) was purchased in the form of ampoules and mixed with sesame oil (5 μg vitamin D + 150 μl sesame oil) to be injected subcutaneously into the target groups (Ca.VD and Ca.Ex.VD) once every 2 days. Other groups (Ca.C and Ca.Ex) except the healthy control group (H.C) were treated with the same volume of sesame oil as a placebo.

### Tumor Volume Measurement and Tissue and Blood Sampling

Tumor volume was measured in two dimensions. The largest dimension of the tumor was considered as the length of the tumor (L), and the other dimension (at 90°) was considered as the width of the tumor (W). At the end of each week, the length and width of the cancerous tumor were measured by a digital caliper (EKO; TURBO, NO: EDC-20), and the tumor volume was calculated according to the following formula [V = 1/2 (L^2^ × W)] (Jones et al., 2010).

To blood sampling and tissue procurement, the animals were first anesthetized by subcutaneous injection of ketamine-xylazine. Blood samples were then taken directly from the animal’s heart (1.5 ml). The blood was then placed into tubes containing EDTA and centrifuged at 3000 *g* for 10 min, and after serum separation, it was kept at −20°C until the final assay. Immediately following blood sampling, the heart was completely removed, and after weighing, it was fixed in buffered formalin (10%) for 24 h. Cardiac tissue was eventually stored at −80°C for further evaluation.

### Total RNA Extraction and Real-Time PCR Analysis

Total RNA was extracted from cardiac tissue using the RNX-PLUS reagent method (SINACLON, Cat. No: EX6101). In brief, after the myocardial tissue has reached 0°C from a frozen state, about 30–50 mg of the cardiac tissue is homogenized using a hand mixer (NS-16010) by adding liquid nitrogen. The homogeneous tissue was then incubated for 5 min at room temperature. Then, 250 μl of chloroform and 1 ml of RNX-PLUS solution were added and incubated for 5 min. The mixture was then centrifuged at 12,000 r/min for 15 min at 4°C. Total RNA was transferred to an RNase-free situation, and 300 μl of isopropanol was added. After several steps of washing and RNA separation, the extracted RNA was read by a spectrophotometer (NanoDrop ND-1000; Thermo Scientific) at 260 nm. RNA quality was assessed by agarose gel electrophoresis (visual assessment of 28:18 s Ribosomal RNA). RNA was aliquoted and stored at −80°C until further analysis.

A PCR Thermal Cycler (BIORAD T100) was used for reverse transcriptase reaction (Easy^TM^ cDNA synthesis kit; Parstous; CatA101161). cDNA synthesis was carried out using 6 μl of total RNA, 100 ng of random hexamers, and 200 units of RNase reverse transcriptase. cDNA samples were stored at −20°C for later analysis. Primer pairs for target genes were obtained from Metabion (^©^Metabion International AG, Germany). All primers were checked for specificity to the genes of interest by Blast analysis. The qPCR method used as an example to assess performance was valid Prime (Table 2).

**TABLE 2:** Primer sequence (5′→3′).

**Gene**	**Forward**	**Revers**
*Pgc-1*α	TGAACTAAGGGATGGCGACT	AAGAAGGCGACACATCGAAC
*Mfn-1*	GTTTTAGTAGACAGCCCAG	GTCCGTGTTCATCAGTGTT
*Drp-1*	TTTGCTAGATGTGCCAGTTCC	ATTACTGCCTTTGGGACGCTG
*Gapdh*	AAATGGTGAAGGTCGGTGTG	GAATTTGCCGTGAGTGGAGT

The real-time thermal cycling system (Corbett 5Plex Rotor-gene 6000; Qiagen) was used for all samples for determination of relative mRNA expression, utilizing the Real Q Plus 2x Master Mix Green without ROX (VIRAGENE; Code: A323402) plus 1 μl of the first-strand cDNA as a template and 0.6 μl of the upstream and downstream PCR primer. The three-step RT-PCR protocol included a 1 min enzyme activation step at 95°C, followed by 40 cycles of 10 s at 95°C, 15 s at 60°C, and 20 s at 72°C. To control for variations, all PCR data were normalized against glyceraldehyde 3-phosphate dehydrogenase (*Gapdh*) expression (Zeljic et al., 2017). The relative mRNA expression levels of the target genes, *Pgc-1*α, *Mfn-1*, and *Drp-1*, were determined by the delta–delta Ct method.

### Total Antioxidant Capacity and 25-Dihydroxyvitamin D Assay

Serum TAC was measured using a commercial kit (by ZB-TAC-48A, Zellbio; Germany). The ferric reducing antioxidant power (FRAP) method was used to measure TAC. This method is based on the ability of plasma or serum ferric to ferrous ion reduction at low PH causing a colored ferric-tripyridyltriazine complex to form. FRAP values were obtained by comparing the absorbance change at 593 nm in test reaction mixtures with those containing ferrous ions in known concentrations. Circulating 25-ihydroxyvitamin D [25(OH) D] concentrations were measured using high-performance liquid chromatography paired with a diode array detector (Benzie and Strain, 1996).

### Statistical Analysis

The normality of data distribution was checked using Shapiro–Wilk test. One-way ANOVA and Tukey’s *post hoc* tests were used to compare differences between groups in the post-test. IBM SPSS software (version 23) was used for data analysis. The statistically significant difference was set at *p* < 0.05. All values are presented as Means ± SD.

## Results

Regarding variables of body weight and cancer tumor volume, measurements were performed in two stages: the beginning of 6-week IET, and the end of the IET program. In the case of other variables (serum levels of vitamin D and TAC, as well as the cardiac tissue of mRNA expression of the gene of *Pgc-1*α, *Mfn-1*, and *Drp-1*); the means of the groups in the post-test were compared.

While in the pre-test there was no significant difference between the study groups in terms of body weight, in the post-test, the lowest body weight was related to the Ca.C group so that the weight of this group was significantly lower than in all three groups receiving the treatments (Ca.VD, Ca.Ex, and Ca.Ex.VD, in all three cases, *p* = 0.001) (Table 3).

**TABLE 3:** Changes in body weight and tumor volume of research groups after 6 weeks of treatment (interval exercise training and vitamin D supplementation) (means ± SE).

		**H.C (*n* = 8)**	**Ca.C (*n* = 8)**	**Ca.VD (*n* = 8)**	**Ca.Ex (*n* = 7)**	**Ca.Ex.VD (*n* = 6)**
Weight (g)	F.W	31.25 ± 1.58	29.13 ± 2.64	30.88 ± 1.36	31.00 ± 2.00	30.67 ± 3.08
	L.W	35.75 ± 2.12	28.38 ± 7.93*	31.63 ± 1.60*	31.29 ± 1.98*	31.87 ± 2.40*
Volume of tumor (mm^3^)	F.W	†	0.044 ± 0.005	0.051 ± 0.002	0.046 ± 0.005	0.047 ± 0.007
	L.W	†	0.279 ± 0.037	0.399 ± 0.030	0.313 ± 0.046	0.263 ± 0.019
	L.W/LW	†	6.34 ± 0.02	7.82 ± 0.02	6.80 ± 0.03	5.60 ± 0.01 *
% T.V.D		†	0%	+ 23%	+ 7%	12%

*P ≤ 0.05, F.W, first week; L.W, last week; L.W/LW, the ratio of the tumor volume from the last week to the first week. † = no tumor; % T.V.D, percentage of tumor volume development; H.C, healthy control group; Ca.C, cancer control group; Ca.VD, cancer with injection of vitamin D group; Ca.Ex, cancer exercise training group; Ca.Ex.VD, cancer exercise training with injection of vitamin D group.

**FIGURE 2:**
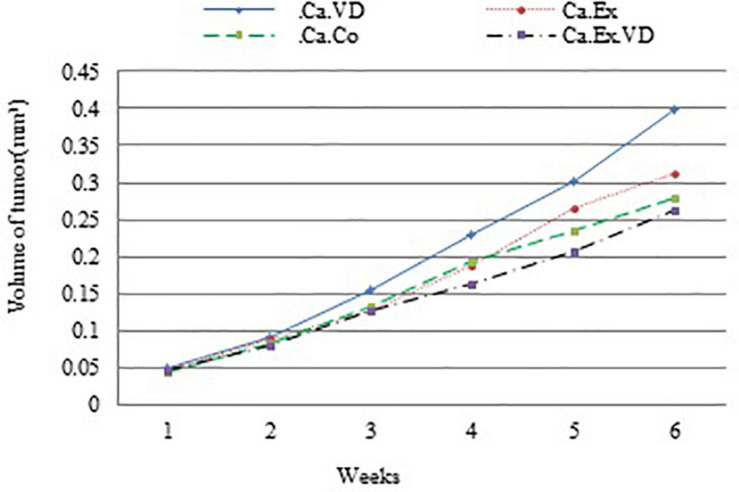
Average tumor volume is plotted relative to the number of weeks during 6 weeks of interval exercise training (IET) and vitamin D supplementation treatment in four cancer groups. Ca.C, cancer control group; Ca.VD, cancer with injection of vitamin D group; Ca.Ex, cancer exercise training group; Ca.Ex.VD, cancer exercise training with injection of vitamin D group.

The ratio of tumor volume growth at the end of the treatment to tumor volume at the beginning of the treatment showed that the lowest tumor growth occurred in Ca.Ex.VD group (*p* = 0.001). In other words, vitamin D treatment alone has increased tumor volume growth by approximately 23%; in contrast, IET with vitamin D has reduced the chance of tumor volume development by 12% (Figure 2 and Table 3).

**FIGURE 3:**
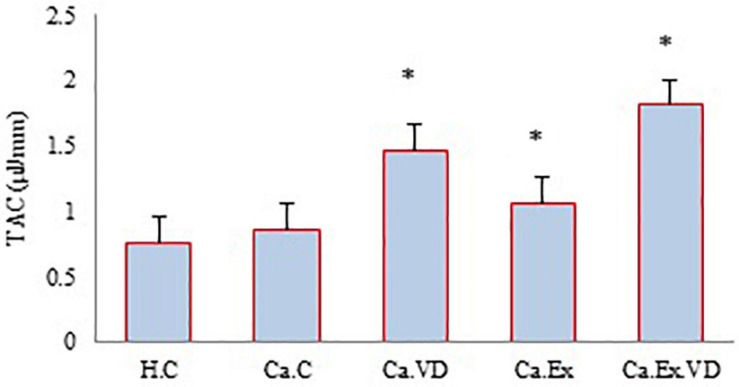
The levels of total antioxidant capacity (TAC) during 6 weeks of interval exercise training (IET) and vitamin D supplementation treatment in all experimental groups. H.C, healthy control group; Ca.C, cancer control group; Ca.VD, cancer with injection of vitamin D group; Ca.Ex, cancer exercise training group, Ca.Ex.VD, cancer exercise training with injection of vitamin D group. Data are presented as mean ± SD. ^∗^Significant level (*P* ≤ 0.05).

Regarding TAC, the results of the present study showed that in the three treatment groups (Ca.VD, Ca.Ex, and Ca.Ex.VD), the levels of TAC were higher than in the cancer control group (Ca.C) (*p* = 0.001) (Figure 3). Indeed, TAC levels were higher in the group that received both vitamin D and IET (Ca.Ex.VD) than in the other treatment groups (Ca.VD and Ca.Ex) (in both cases, *p* = 0.001). The use of both IET and vitamin D treatments has synergistic effects on TAC.

**FIGURE 4:**
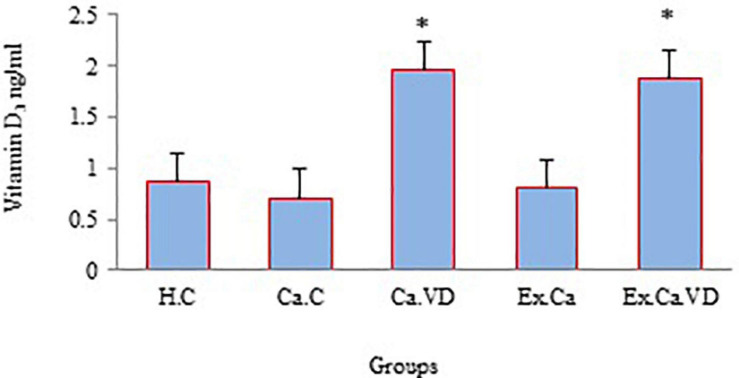
The levels of vitamin D during 6 weeks of interval exercise training (IET) and vitamin D supplementation treatment in all experimental groups. H.C, healthy control group; Ca.C, cancer control group; Ca.VD, cancer with injection of vitamin D group; Ca.Ex, cancer exercise training group; Ca.Ex.VD, cancer exercise training with injection of vitamin D group. Data are presented as mean ± SD. ^∗^Significant level (*P* ≤ 0.05).

In the case of vitamin D, the findings of the current study indicate that the serum levels of this vitamin were higher in the post-test of groups Ca.Ex.VD and Ca.VD compared to groups Ca.Ex (*p* = 0.001 and *p* = 0.001) and Ca.C (*p* = 0.001 and *p* = 0.001) (Figure 4). In other words, groups that received vitamin D supplementation (alone or in combination with IET) were more eligible.

**FIGURE 5:**
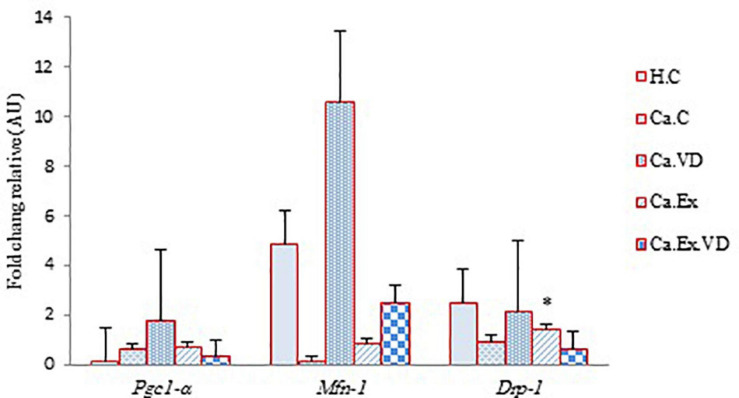
The effect of 6 weeks of interval exercise training (IET) and vitamin D supplementation on *Pgc-1*α, *Mfn-1*, and *Drp-1* genes expression in five control and experimental groups. H.C, untrained healthy control group; Ca.C, cancer control group; Ca.VD, cancer with injection of vitamin D group; Ca.Ex, cancer exercise training group; Ca.Ex.VD, cancer exercise training with injection of vitamin D group. Data are presented as mean ± SD. ^∗^Significant level (*P* ≤ 0.05).

The results of the present study illustrate that there was a significant difference between the groups in terms of cardiac tissue mRNA levels expression of *Pgc-1*α, *Mfn-1*, and *Drp-1* genes (for all three genes, *p* = 0.001). The *post hoc* test showed that the expression of these genes in the vitamin D supplementation group (Ca.VD) was higher than in other groups (Figure 5). In the other words, elevated cardiac mRNA levels of expression of *Pgc-1*α, *Mfn-1*, and *Drp-1* genes are not due to IET, but it is due to the consumption of vitamin D. In fact, it can be inferred from the results that vitamin D treatment alone led to a higher of the expression of all three genes in cardiac tissue, while a lower of the expression of genes in heart tissue occurred through IET with vitamin D except *Mfn-1* gene.

## Discussion

The findings of this study demonstrate that the presence of a tumor in the body of mice alone results in losing weight without applying IET or vitamin D so that the most reduction body weight was seen in the cancer control group than in all three groups receiving the treatments. IET and/or vitamin D almost have prevented the loss of weight and in particular muscular weakness in mice. In line with this work in the recent study, it is indicated that the combination of treatments of HIIT and saffron supplementation affects weight loss and cachexia control in mice BC modeling (Ahmadabadi et al., 2020). Weight loss can depend on the type of cancer; BC or prostate cancer patients are less susceptible to weight loss. As well, loss of weight is associated with changes in metabolic hormones such as insulin, leptin, and cortisol (Hojman et al., 2018), which were not examined in this study and it was one of the limitations of our study.

Moreover, the comparison of the ratio of tumor volume growth displayed that the lowest tumor growth occurred through IET along with vitamin D supplementation not alone. This indicates the synergistic effect of IET and vitamin D supplementation in controlling tumor volume development. While vitamin D treatment alone has increased tumor volume growth by approximately 23%; in contrast, IET with vitamin D has reduced the chance of tumor volume development by 12%. Our results are consistent with a study on the effect of long-term another form of vitamin D supplementation that was demonstrated the role of l,25(OH)_2_D in preventing tumorigenesis through inhibiting oxidative stress and DNA damage so that 1,25(OH)2D3 arrested spontaneous tumor expansion, indicating that this suppressor effect prevents the progression of tumorigenesis and thus supplies a mechanistic foundation for 1,25(OH)2D3 to prevent tumorigenesis in the organism (Chen et al., 2018). Recently, a meta-analysis study reported that physical exercise leads to a decrease in tumor size in animals (Eschke et al., 2019). Various mechanisms such as altering hormones (androgen, IGF-1, insulin, circulating, and leptin) reduced tumor volume, and reformed antioxidative defense and immune care (Mahabir et al., 2018). As well as, in line with our work, the earlier study demonstrated the effects of regular exercise training in controlling tumor development and diminishing systemic levels of risk factors such as those confirmed for BC (Hojman et al., 2018). Besides, tumor-related vital metabolic abnormalities include an imbalance between glycolysis and oxidative phosphorylation, dysregulation of metabolic enzymes, and alterations of gene expression levels linked to tumorigenesis that are influenced by the vitamin D status (Wu et al., 2019). According to the result of current research, less expression of the main factor of mitochondrial biogenesis, *Pgc-1*α, may be related to the volume of tumor development, thus suggesting that is being a mechanistic basis to diminish tumor volume growth following the decline of expression of *Pgc-1*αmRNA in combination of IET and vitamin D. So, the findings of this study are consistent with previous research that demonstrates the delay of possible tumor development and smaller-sized tumors (Malicka et al., 2015; Alvarado et al., 2016; Faustino-Rocha et al., 2016, 2017; Sturgeon et al., 2017).

Furthermore, the results of this work indicate that treatments lead to the elevation of the levels of TAC. The use of both exercise and vitamin D treatments had efficient synergistic effects on TAC, although each of the treatments had such outcomes independently. Since the number of oxidative stress increases in the tumorigenesis state, the maintenance of a less level of ROS is critical for cancer cells to avoid oxidative stress (Zhong et al., 2019). In line with our work, researchers reported that acute exercise training also for 3 or 5 days illustrated the preservation of myocardium and reinforcement of fatigue resistance of the diaphragm by improving the antioxidant status (Child et al., 1998; Demirel et al., 2001). The prior study has shown to elevate the TAC in human blood even after a single bout of exercise (Berzosa et al., 2011). Most of the results are consistent with the idea that exercise may play a beneficial role in protecting against BC and CVD due to its potential to increase antioxidant defense mechanisms through a redox-sensitive pathway. Improved serum TAC can indicate the body’s defensive response and capability in preventing exercise-induced oxidative damage (Ficicilar et al., 2006). Moreover, recent developments in vitamin D research indicate that this vitamin is one of the key controllers of systemic inflammation, oxidative stress, and mitochondrial respiratory function. Vitamin D may be a strong antioxidant that comforts balanced mitochondrial activities, preventing oxidative stress-related protein oxidation, lipid peroxidation, and DNA damage (Ya et al., 2015; Wimalawansa, 2019). Therefore, most of the results of the studies are consistent with the findings of our work and we also found the effect of each treatment and their combination in improving the TAC which is likely beneficial to cardioprotective and BC.

As well as there was a significant difference between cardiac tissue mRNA level of expression of *Pgc-1*α, *Mfn-1*, and *Drp-1* genes. The results exposed that the vitamin D supplementation results in more expression of genes in cardiac tissue in mice. Previous findings have shown the effect of vitamin D treatment on the global transcriptomic profile and more regulation of 3.2-fold genes in women than in men (Atoum and Alzoughool, 2017). The results of our work show that vitamin D treatment results in a greater expression of genes in cardiac tissue in mice so that these mice practically exhibit more treadmill running capacity and increased cardiac function during exercise. So, we could detect a stronger effect of vitamin D supplementation on gene expression in female mice with BC modeling. Recently, a study showed that aerobic exercise was an effective stimulator to induce the expression of PGC-1α and VEGFA and hypoxia condition also, but their combination did not have a synergistic effect (Rahimifardin et al., 2020). In our work, vitamin D led to an elevation of *pgc-1*α expression but in combination with IET did not have a synergistic effect. Nevertheless, it was beneficial to BC treatment and inhibition of tumor development and through diminish of *pgc-1*α also prevents angiogenesis and metastasis of cancer cells to the heart muscle. It is worth noting that paradoxically, PGC-1α against various treatments may have different effects that not only by fewer levels of expression tumor-suppressive and inhibition of angiogenesis effects but also by greater rates of expression led to antioxidant defense effects which depending on the circumstances both are useful in BC and CVD patients. As in our work the less mRNA level of expression of *Pgc-1*α gene by the combination of IET and vitamin D likely results in the greater reduction in volume tumor growth or prevents from more tumor development. On the contrary, vitamin D treatment without exercise treatment in the cancer group (tumor) has increased the mRNA levels of expression of *pgc-1*α, *Mfn-1*, and *Drp*-1 genes that indicated the activation of mitochondrial biogenesis pathways and mitochondrial fusion, and fission process in the mitochondrial dynamics, respectively. According to a previous study, the elevation of nuclear PGC-1α expression following aerobic interval training represses pathological altering by the elevation of nuclear PGC-1α expression and preventing mitochondrial dysfunction following myocardial infarction (MI) in rats (Jiang et al., 2014). In line with noted work, vitamin D treatment also prevented mitochondrial dysfunction in BC and CVD. Besides, the expression ratio of *the Mfn1* gene to the *Drp1* gene is fivefold higher (10.56 vs. 2.11), thus this challenge in favor of fusion. Consequently, the increase of mitochondrial fusion induces increased apoptosis (Chang et al., 2019). In agreement with that, the increase of *Mfn-1* is due to the increased apoptosis during tumorigenesis as well as the establishment of mitochondrial network extension (Kemi et al., 2008). As well as, the Mfn-1 overexpress by vitamin D treatment with/without IET treatment, it could induce increased mitochondrial fusion, subsequently prevented the migration and invasion of BC cells, and has activated the apoptosis pathway.

Researches revealed that the combination of exercise and daidzein synergistically excited and redistributed natural killer (NK) cells through an increase of regulating the level of epinephrine and IL-6, co-treatments also induces apoptosis in cancer cells via Fas/FasL-initiated mitochondrial apoptosis signaling pathway (Wang et al., 2020). Current evidence supports the benefits of the combination of exercise training and vitamin D for improving BC risk factors linked to CVD prognosis. In the current work, IET without vitamin D treatment could not have been such a positive effect on the improvement of mitochondrial fusion and expansion of mitochondrion network in cardiac tissue in BC mice, while it induces better in *Drp-1* gene expression. This indicates the activation of the mitochondrial fission pathway than infusion and even mitochondrial biogenesis, likely due to increased oxidative stress in both cancerous tumors and doing exercise training in mice. Although fission in turn is an important stage in the liberation of cytochrome C and consequently, improved apoptosis (Estaquier and Arnoult, 2007), fission-dependent apoptosis appears to be context and cell-type-dependent (Kashatus, 2018). So we conclude that the *pgc-1*α gene as a key regulator of energy metabolism will make a useful decision for improving a patient or the success of an athlete. Increasing the body’s antioxidant defensive system against oxidative stress caused by active species in cancer cells and decreasing the expression of the *pgc-1*α gene prevent angiogenesis and metastasis of cancer cells to the heart muscle and other parts of the body.

## Conclusion

In conclusion, synergistic responses lead to controlling body weight, tumor volume growth, and improving TAC. Besides, it occurred diminishing the levels of *pgc-1*α gene expression as suppressive of the tumor and repressive of the angiogenesis that a challenging issue and warranted further study. As well they led to elevating *Mfn-1* gene expression that helps to the evolution of the mitochondrial network to optimizing energy metabolism through activation mitochondrial fusion pathway. Co-treatments of IET and vitamin D also showed a reduction of the levels of *drp-1* gene expression release of cytochrome C and improvement of fission-dependent apoptosis. Of course, this type of fission-dependent apoptosis depends on the type of tissue and cell type. IET and vitamin D supplementation could be potential anti-cancer agents in treatment BC in humans and preventing CVD subsequent BC. It is recommended to be carried out further studies on mitochondrial dynamics and biogenesis in cardiac muscle of patients with BC to preventing CVD.

## Data Availability Statement

The raw data supporting the conclusions of this article will be made available by the authors, without undue reservation.

## Ethics Statement

The project was found to be in agreement with the ethical platform and the national norms and standards for conducting medical research in Iran (Approval ID: IR.UOK.REC.1397. 026).

## Author Contributions

AJ and DS-V designed the study and analyzed and interpreted the data. AJ and NK collected the data. AJ drafted the manuscript. DS-V, AJ, and FK revised the manuscript. All authors contributed to the article and approved the submitted version.

## Conflict of Interest

The authors declare that the research was conducted in the absence of any commercial or financial relationships that could be construed as a potential conflict of interest.

## Footnotes

1 Dulbecco’s modified Eagle’s medium
