# Supplementary figures and images for: Synergistic Effects of Exercise Training and Vitamin D Supplementation on Mitochondrial Function of Cardiac Tissue, Antioxidant Capacity, and Tumor Growth in Breast Cancer in Bearing-4T1 Mice
Source: Front Physiol. 2021 Apr 13;12:640237. doi: 10.3389/fphys.2021.640237 (PMC8076802; doi:10.3389/fphys.2021.640237)

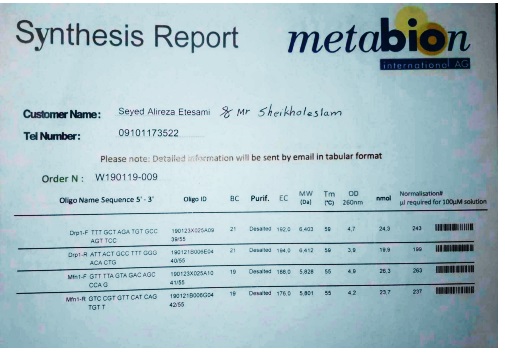

Supplement: Supplementary file 1 [file Image_1.JPEG]

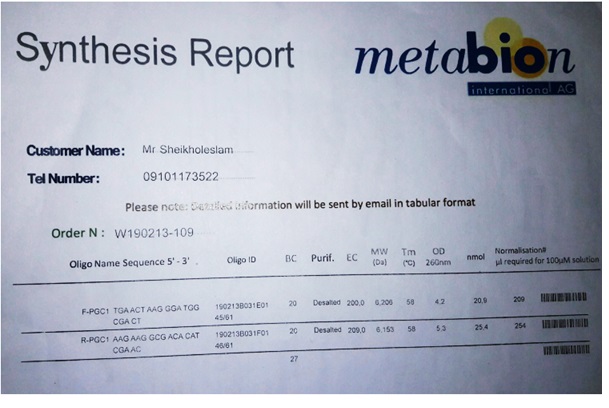

Supplement: Supplementary file 2 [file Image_2.JPEG]
